# Supplementary material for: Conversion of Hyperpolarized [1-13C]Pyruvate in Breast Cancer Cells Depends on Their Malignancy, Metabolic Program and Nutrient Microenvironment
Source: Cancers (Basel). 2022 Apr 6;14(7):1845. doi: 10.3390/cancers14071845 (PMC8997828; doi:10.3390/cancers14071845)
Supplement: Supplementary file 1 [file cancers-14-01845-s001.zip › cancers-1635683-supplementary.pdf]

Conversion of Hyperpolarized [1-^{13}C]Pyruvate in Breast Cancer Cells Depends on their Malignancy, Metabolic Program and Nutrient Microenvironment

Martin Grashei, Philipp Biechl, Franz Schilling, and Angela M. Otto

Supplementary Material

Content:

Table S1: Intracellular concentrations of lactate and pyruvate in MCF-7 and MDA-MB231 cells.

Table S2: Calculations of LDH_{eff}.

Figure S1: Example of water fall plots of dynamic hyperpolarized ^{13}C-MRS spectra.

Figure S2. Western blots of protein expression of MCT1 in MCF-7 and MCT4 in MDA-MB231.

Figure S3. Intracellular pyruvate and lactate levels in MCF-7 and MDA-MB231 cells in different glucose/glutamine conditions as determined by GC-MS.

Table S1: **Intracellular concentrations of lactate and pyruvate in MCF-7 and MDA-MB231 cells.***

A. MCF-7

**combinations**		ratio				calculated
glucose mM	glutamine mM	**lactate mM**	*dev.*	**pyr/lac**	**lac/pyr**	**pyruvate µM**
1	0.1	**1.50**	*0.69*	0.021	46.5	32.2
2.5	0.1	**2.90**	*1.25*	0.015	65.8	44.0
1	1	**1.33**	*0.69*	0.015	68.3	19.4
2.5	1	**1.24**	*0.58*	0.023	43.3	28.7
25	4	**9.92**	*7.13*	0.020	48.8	**203.2**

B. MDA-231

**combinations**		ratio				calculated
glucose mM	glutamine mM	**lactate mM**	*dev.*	**pyr/lac**	**lac/pyr**	**pyruvate µM**
1	0.1	8.36	0.93	0.0043	234.0	35.7
2.5	0.1	13.06	1.07	0.0056	179.6	72.7
1	1	5.67	0.8	0.0045	222.1	25.5
2.5	1	4.29	0.46	0.0071	140.9	30.5
25	4	12.46	1.72	0.0027	376.3	33.1

* Lactate levels per sample were determined using the LDH-based assay (Material and Methods). These were used for calculating intracellular concentrations based on a cellular volume of 2 pL /cell and the cell number in the sample [lac_{LDH}]. For determining the pyruvate concentrations, the ratio of the relative levels of pyruvate/lactate, as determined by GC-MS relative to norvaline, were multiplied with the respective lac_{LDH} concentrations.

Table S2: **Calculations of effective LDH activities (LDH_{eff})***

A. MCF-7 cells

**culture conditions**		**NAD+** (n=2)		**NADH** (n=2)		**lactate** (n=3)		**pyruvate** (Table S1)	**LDH activity** (V_{max})	***effective* LDH activity** (LDH_{eff})
glucose mM	glutamine mM	**µM**	*dev.*	**µM**	*dev.*	**mM**	*dev.*	**µM**	µmol/min/ 10e6 cells*	µmol/min/ 10e6 cells*
1	0.1	**607**	*73*	**95**	*29*	**1.50**	*0.69*	**32.2**	0.175	**0.052**
2.5	0.1	**771**	*142*	**143**	*30*	**2.90**	*1.25*	**44.0**	0.167	**0.054**
1	1	**596**	*73*	**105**	*15*	**1.33**	*0.69*	**19.4**	0.135	**0.030**
2.5	1	**624**	*13*	**94**	*8*	**1.24**	*0.58*	**28.7**	0.144	**0.052**
25	4	650**	*71*	*110***	*17*	**9.92**	*7.13*	**203.2**	**0.199**	**0.114**

B. **MDA-MB231 cells**

**culture conditions**		**NAD+** (n=2)		**NADH** (n=2)		**lactate** (n=3)		**pyruvate** (Table S1)	**LDH activity** (V_{max})	***effective* LDH activity** (LDH_{eff})
glucose mM	glutamine mM	**µM**	*dev.*	**µM**	*dev.*	**mM**	*dev.*	**µM**	µmol/min/ 10e6 cells*	µmol/min/ 10e6 cells*
1	0.1	**452**	*164*	**2.1**	*1*	**8.36**	0.93	**32.1**	0.187	**0.043**
2.5	0.1	**601**	*68*	**97.2**	*32*	**13.06**	1.07	**65.7**	0.119	**0.045**
1	1	**519**	*22*	**23.8**	*7*	**5.67**	0.80	**21.2**	0.197	**0.035**
2.5	1	**625**	*78*	**65.4**	*5*	**4.29**	0.46	**27.0**	*nd*	*nd*
25	4	**549****	*42*	***81****	*16*	**12.46**	1.72	**26.6**	0.155	**0.034**

*Values of intracellular substrate concentrations and potential LDH activities (V_{max}) were used to calculate the effective LDH activities (LDH_{eff}) of cells from MRS measurements. For simplification and calculations using the Michaelis-Mention equation, it was assumed that the K_M for pyruvate is 100 µM (see Results, Fig. 3 (Goto et al., 2016) and that NADH was already enzyme-bound, pyruvate thus being the rate-limiting component.

** Values of NAD^+ and NADH averaged from respective values which are apparently similar in the different glucose/glutamine conditions

Reference for Table 2:

Goto, T., Sugawara, K., Nakamura, S., Kidokoro, S. I., Wakui, H., & Nunomura, W. (2016). Enzymatic and thermodynamic profiles of a heterotetramer lactate dehydrogenase isozyme in swine. Biochemical and Biophysical Research Communications, 479(4), 860-867. doi:10.1016/j.bbrc.2016.09.118

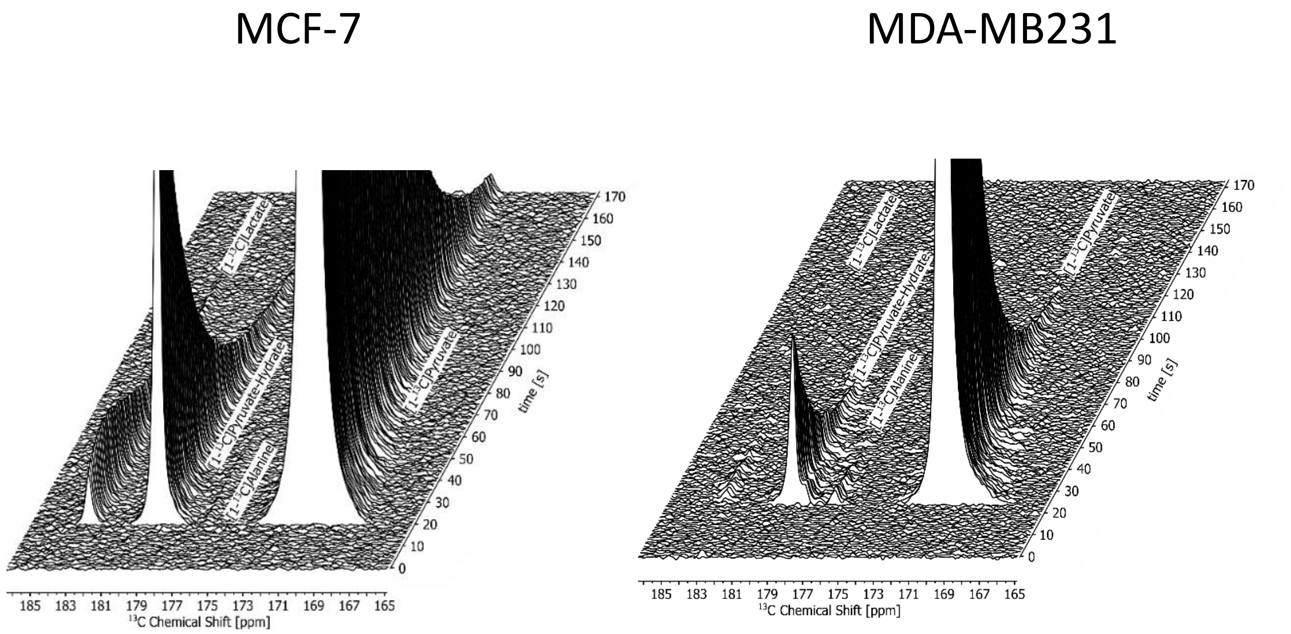

Figure S1: Example of water fall plots of dynamic hyperpolarized ^{13}C-MRS spectra of a 2 min-acquisition upon injection of 1 mM [1-^{13}C] into MCF-7 and MDA-MB231 cell suspensions. Cells had been incubated 72 h in medium containing 2.5 mM glucose and 0.1 mM glutamine.

MCF-7

glucose mM	1	1	2.5	2.5	1	1	2.5	2.5	
glutamine mM	0.1	0.1	0.1	0.1	1	1	1	1	
marker									marker

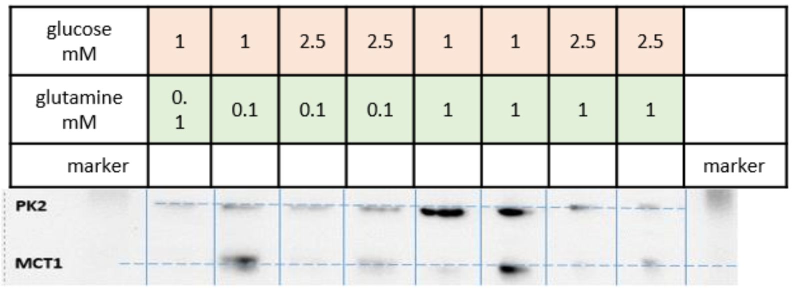

MDA-MB231

glucose mM	1	1	2.5	2.5	1	1	25	25	
glutamine mM	0.1	0.1	0.1	0.1	1	1	4	4	
marker									marker

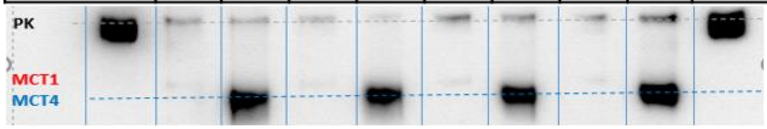

Figure S2. Western blots of protein expression of MCT1 in MCF-7 and MCT4 in MDA-MB231, relative to pyruvate kinase (as marker) in variable glucose / glutamine conditions.

Shown are representative blots of 2-3 similar experiments for each cell line. The unusual high PK signal in MCF-7 cells may be a technical deviation, as it is not observed in other blots for the same glucose/glutamine conditions.

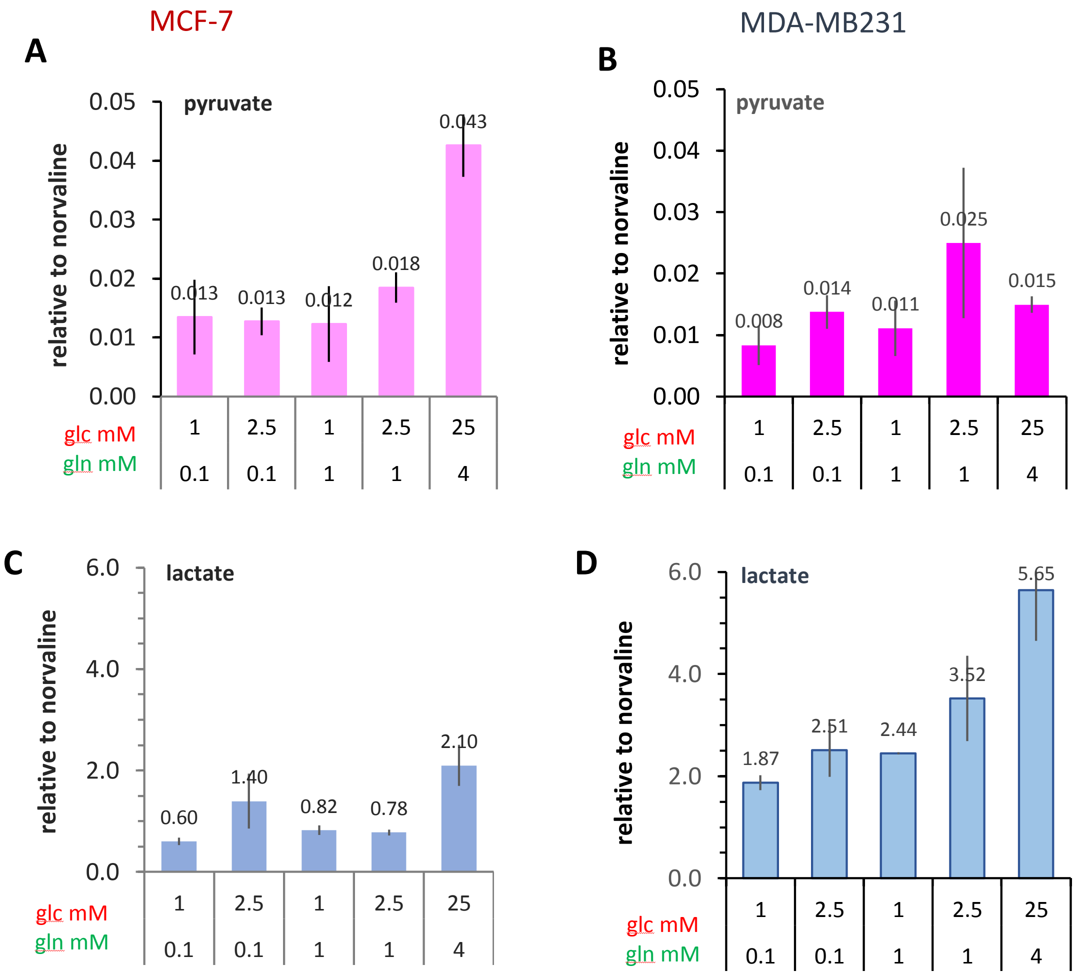

Figure S3. Intracellular pyruvate and lactate levels in MCF-7 and MDA-MB231 cells in different glucose/ glutamine conditions as determined by GC-MS.
Cells were incubated for 2 h with medium containing the indicated [U-$^{13}C_6$]glucose and glutamine concentrations and prepared for metabolite analyses by GC-MS (Material and Methods). Following chromatographic separation, metabolites were quantified relative to the GC-reference compound norvaline. Shown are averages and s.e.m. of two experiments for (A, C) MCF-7 and (B, D) MDA-MB231-MB231
